# Supplementary material for: Female vocalizations predict reproductive output in brown-headed cowbirds (Molothrus ater)
Source: PLoS One. 2018 Dec 26;13(12):e0202067. doi: 10.1371/journal.pone.0202067 (PMC6306164; doi:10.1371/journal.pone.0202067)
Supplement: S1 Table — (DOCX) [file pone.0202067.s002.docx]

Supplementary Information

Sup 1. Correlations in the proportion of response chatters across years for females who maintained paired males across years (same male) and those who switched paired males (different male)

| Paired male | N | Coefficient | P value | 95% CI |
| --- | --- | --- | --- | --- |
| Same male | 8 | *Rho* = 0.66 | 0.7 | 0.01 – 1.0 |
| Different male | 20 | *Rho* = 0.56 | 0.009 | 0.15 -0.94 |

Supplemental Materials 1. Correlation between females who were paired with the same male across the 2011 to 2012 breeding season, and females who paired with different males across breeding seasons. While both were positively correlated, a significant correlation was observed in the females with different paired males.
